# Preface to the special issue “Current Status and Future Prospects for the Development of Crop Varieties and Breeding Materials Using Genome Editing Technology”

**DOI:** 10.5511/plantbiotechnology.23.0000p

**Published:** 2023-09-25

**Authors:** Masahiro Nishihara, Toshiya Muranaka

**Affiliations:** 1 Iwate Biotechnology Research Center, Kitakami, Iwate 024-0003, Japan; 2 Department of Biotechnology, Graduate School of Engineering, Osaka University, Suita, Osaka 565-0871, Japan; 3 Institution for Open and Transdisciplinary Research Initiatives, Osaka University, Suita, Osaka 565-0871, Japan

Genome editing technology has evolved significantly in recent years. Since the groundbreaking work conducted by Gasiunas et al. (2012) and Jinek et al. (2012), it has become a powerful and versatile tool, not only for fundamental research but also for investigating the mechanisms of gene function and its application in various fields. Several methods based on CRISPR technology have emerged, including base editing (Komor et al. 2016; Nishida et al. 2016) and prime editing (Anzalone et al. 2019). While some of these techniques are still under evaluation for their applicability in numerous crops, they hold immense promise.

There are excellent reviews available on genome editing, encompassing not only model plants but also practical crops such as cereals, vegetables, fruits, and flowers (Gao 2021; Naik et al. 2023; Pramanik et al. 2021). To facilitate the practical use of genome-edited plants, it is crucial to establish foreign-DNA-free lines. Various strategies for null segregant production have been proposed (reviewed in detail by Bhattacharjee et al. 2023), including crossing or direct transformation of primary transformants. Additionally, methods involving Ribonucleoproteins (RNPs), protoplasts, pollen, or reproductive techniques have been explored (Toda et al. 2022). Recently, a grafting-mediated genome editing system was also reported (Yang et al. 2023), adding to the array of available techniques.

In this special issue, we aim to explore and discuss the latest advancements in genome editing technology, its applications, for the development of crop varieties and breeding materials.

## The tools used for genome editing

The field of genome editing relies on three primary technologies: zinc-finger nucleases (ZFNs) introduced by Kim et al. (1996), transcription activator-like effector nucleases (TALENs) pioneered by Christian et al. (2010), and the clustered regularly interspaced short palindromic repeats (CRISPR)-CRISPR-associated protein 9 (Cas9) system developed by Gasiunas et al. (2012) and Jinek et al. (2012). Among these, the most recent advancement, the CRISPR/Cas9 system, has become the go-to choice for genome editing due to several key advantages, including high efficiency, precision, ease of vector construction, and minimal off-target effects when compared to TALENs and ZFNs.

As of September 14, 2023, a search on PubMed indicates the popularity and extensive use of these genome editing technologies, with 862 hits for Zinc finger nuclease genome editing, 1,413 for TALEN, and a staggering 13,154 for CRISPR Cas9. This overwhelming disparity in the number of publications highlights the dominance of the CRISPR/Cas9 system in genome editing research and applications.

## Production of enhanced crops through genome editing

Genome editing has emerged as a revolutionary tool in crop improvement, offering a versatile means to address diverse breeding targets. The challenges faced in crop breeding can vary significantly depending on the species, particularly in cases involving highly heterozygous and complex polyploid genomes. In such instances, achieving elite crop lines within short timeframes becomes a formidable task, necessitating innovative approaches.

Genome editing systems have primarily focused on SDN-1 editing, predominantly gene knockout of specific target genes, and have demonstrated success in introducing small insertion, deletion and substitution via non-homologous end joining (NHEJ). Moreover, researchers have expanded their focus to include promoter and intron regions as mutagenesis targets, resulting in more precise and controlled genetic modifications. The emergence of base editing, a highly accurate system that enables the modulation of enzyme activity, holds great promise and has been the subject of numerous recent reviews and studies (Azameti and Dauda 2021; Verma et al. 2023).

Across the globe, the development of genome-edited crops is advancing at a rapid pace. This progress is driven by the utilization of novel genome editing techniques, some of which are discussed here. Additionally, examples from recent reviews further illustrate the widespread adoption and evolution of genome editing technologies.

In this context, some genome-edited crops are directly employed as cultivars, while others serve as valuable materials for traditional crossbreeding programs. The flexibility and precision offered by genome editing provide plant breeders with unprecedented tools to address various breeding challenges and create improved crop varieties.

## MAFF commissioned project: Advancing crop breeding through genome editing

In 2019, a pioneering research endeavor was initiated as a 5-year project supported by the MAFF (Ministry of Agriculture, Forestry and Fisheries) Commissioned Project. The primary goal of this study, titled “Development of New Varieties and Breeding Materials in Crops by Genome Editing”, is to create breeding materials for various crops utilizing genome editing technology. Leading this ambitious initiative are the project representatives, Dr. Muranaka overseeing the comprehensive project (grant no.: JP19191192) and Dr. Nishihara responsible for the individual project (grant no.: JP19190722).

The research consortium comprises a diverse group of experts, including those from academia, research institutes, and companies, all dedicated to advancing crop breeding across a range of plant species. These include crops such as, potato, wheat, soybean, rice, bell pepper, onion, gentian, lily, and more. The ultimate objective over the course of these five years is to produce cultivars and breeding materials through genome editing, with some of the resulting genome-edited plants already confirmed as null-segregants and under cultivation in experimental field trials, setting the stage for potential future commercialization.

This initiative has also yielded critical advancements in fundamental technologies aimed at enhancing genome editing efficiency in plants. Innovations include virus-mediated genome editing (as reviewed by Oh et al. 2021), the application of Ribonucleoproteins (RNPs) (as detailed by Zhang et al. 2021), and the iPB method (Hamada et al. 2017), among others. A noteworthy highlight is the exploration of Cas3 technology (Morisaka et al. 2019) to crop plants, showcasing the project’s commitment to pushing the boundaries of genome editing.

Confirmation of null segregants is crucial, and this has been achieved through Southern blot analysis and PCR, with next-generation sequencing methods like *k*-mer analysis (Itoh et al. 2020: Yasumoto and Muranaka 2023) proving invaluable for assessing the genetic integrity of edited crops.

Some of the genome-edited breeding materials being developed in this project are as follows,

Potatoes that do not sprout during storage, reducing costs and losses during storage, wheat that is resistant to red mold and can control fungal toxin contamination and reduce pesticide use, soybean with reduced allergenic components, and gentian with double flowers and improved flower endurance.

As described above, genome-edited strains have been created to develop crops with traits that confer significant benefits that are difficult to produce with conventional breeding techniques.

In the case of both potato and wheat, starting in 2021, we have expanded our evaluations beyond greenhouse trials to include assessments in actual field conditions. This transition is essential for gaining a comprehensive understanding of how the edited traits perform in real-world agricultural settings, ensuring the practical viability of these improvements.

This special issue provides reviews of research and original papers related to this project.

## Cultivation of genome-edited crops in the general field

The cultivation of genome-edited crops in open fields is subject to regulatory procedures, following the rules of the Cartagena Protocol. If these crops contain genome-edited tools, they must be treated as genetically modified organisms (GMOs). Practical use of such crops in Japan requires approval from relevant ministries. Notably, in 2019, a comprehensive policy regarding genome-edited organisms was announced by multiple ministries in Japan, including the Ministry of Health, Labour and Welfare, the Ministry of Education, Culture, Sports, Science and Technology, the Ministry of Economy, Trade and Industry, and the Ministry of Agriculture, Forestry, and Fisheries. Detailed information on these policies can be found in (Tabei and Muranaka 2020). In essence, genome-edited organisms classified as SDN-1, which do not contain foreign DNA sequences (null segregants), may not be considered GMOs if information about the edited organisms is submitted to regulatory authorities. While submission is not obligatory, it is highly recommended for ensuring consumer understanding of genome-edited products.

## Brief introduction of each paper in this special issue

We will discuss three review papers and five original research papers that explore the applications and advancements in crop genome editing, particularly focusing on *Solanum* species, gentian, soybean, potato, and wheat.

### Reviews

Akiyama et al. (2023) (Recent Advances in Steroidal Glycoalkaloid Biosynthesis in the Genus *Solanum*)
  Steroidal glycoalkaloids (SGAs) are specialized metabolites found in *Solanum* species, including toxic substances in crops like tomato, potato, and eggplant. This review discusses recent studies on SGA biosynthesis, including the formation of steroidal aglycones and glycosylation at the C-3 hydroxy group. It also highlights the potential of genome editing to develop non-toxic potatoes by leveraging these findings.
Sim et al. (2023a) (Recent Advances in the Improvement of Soybean Seed Traits by Genome Editing)
  Soybean seed traits are crucial for food, forage, and industrial applications. This review covers the advancements in genome editing for soybeans, focusing on the CRISPR/Cas9 system. Topics include modifications of fatty acid composition, protein content, flavor, size, and seed-coat color. Various transformation platforms for genome editing in soybeans are also discussed.
Kusano et al. (2023) (Efficiency of Potato Genome Editing)
  To efficiently edit the tetraploid genome of potatoes, the CRISPR/dMac3-Cas9 system was developed. Using three guide RNAs (gRNAs), the study achieved high mutation rates in the granule-bound starch synthase (GBSS) and starch branching enzyme (SBE) genes, resulting in functionally defective mutants. The paper also discusses the influence of the number of gRNAs on mutagenesis efficiency.

### Original Papers

Umemoto et al. (2023) (Integrated Gene-Free Potato Genome Editing)
  This original paper presents a method for genome editing in potatoes using transient TALEN and regeneration-promoting gene expression. The study successfully obtained genome-edited potatoes with positive selection and confirmed the absence of foreign DNA sequences, providing a promising approach for crop improvement.
Ohnuma et al. (2023) (Peculiar Properties of Tuber Starch in Potato Mutants Lacking *GWD1*)
  This study focuses on the potato GWD1 gene, involved in starch phosphorylation. Mutants lacking GWD1 exhibited unique phenotypic traits, including growth retardation and altered starch properties, such as increased amylose content and reduced phosphorus levels. These findings shed light on the physiological roles of GWD1 in tuber starch formation and its impact on potato traits.
Nishihara et al. (2023) (Efficient Double-Flowered Gentian Plant Production using the CRISPR/Cas9 System)
  Japanese cultivated gentians are valued ornamental flowers, but they mostly exhibit single-flower types. This paper explores the use of CRISPR/Cas9 genome editing to increase double-flowered gentians by targeting the *AGAMOUS* (*AG1*) floral homeotic gene. The study successfully produced double-flowered gentians and emphasized the potential for future breeding programs.
Kishi-Koboshi et al. (2023) (Optimizing Genome Editing Efficiency in Wheat)
  Wheat is a critical crop, and this paper explores strategies to improve Cas9-induced mutation rates in wheat plants. The study found that using the TaU6 promoter for single guide RNA expression and applying heat treatment during transformation significantly enhanced mutation rates. This research lays the groundwork for improving gene function analysis and crop improvement in wheat.
Sim et al. (2023b) (Site-Directed Mutagenesis of Soybean PEAPOD Genes)
  This research investigates the effects of using the CRISPR/Cas9 system to mutate soybean PEAPOD (PPD) genes, resulting in altered tissue developmental transitions. The study demonstrates that complete knockout of PPD has a negative impact on soybean organogenesis and provides insights into the relationship between PPD and other soybean genes.

In summary, these review and research papers collectively demonstrate the remarkable progress made in crop genome editing across various plant species. Genome editing technologies offer promising avenues for enhancing crop characteristics, improving agricultural productivity, and addressing the challenges of global food security. The studies discussed in this summary contribute valuable insights and methodologies to the field of crop biotechnology and genetic improvement.

## Future perspectives

In our project, some breeding materials developed using genome editing technology have been successfully cultivated in the field for the evaluation of obtained traits. However, further advancements in crop improvement through genome editing necessitate the development of reliable editing systems and efficient null-segregant production methods. While major crops have been the focus of genome editing studies, there remain minor crops that could benefit from these technologies. Additionally, efforts to gain wider public acceptance and suitable regulation of genome-edited products are ongoing. It is imperative to substantiate the safety and benefits of these products through rigorous scientific evidence. The importance of intellectual property rights and patent considerations in this context should also be acknowledged. Currently, GABA-enriched tomatoes are the only commercially available genome-edited crop in Japan; however, we anticipate the release of successive products utilizing genome editing technology in the near future.
